# Severity and Morphological Characteristics of Anaemia Among 6 to 59 Months Old Children in Temeke, Dar es Salaam-Tanzania: Clinics Based Cross Sectional Analysis

**DOI:** 10.24248/eahrj.v8i2.780

**Published:** 2024-06-26

**Authors:** Salha Ally Omary, Florence Salvatory Kalabamu, Maulidi Rashidi Fataki, Shani Shamsi Salum, Ummulkheir Hamid Mohamed, Joseph Gasper Kimaro, Kelvin Melkizedeck Leshabari

**Affiliations:** a Department of Pediatrics/Child Health, Hubert Kairuki Memorial University, Dar es Salaam Tanzania; b Department of Pediatrics/Child Health, Temeke Regional Referral Hospital, Dar es Salaam Tanzania; c NUTritional REsearch iNTer-Collaboration (NUTRENT) Group, Registered Trustees of Ultimate Family Healthcare, Dar es Salaam, Tanzania; d Ageing Research Group, Registered Trustees of Ultimate Family Healthcare, Dar es Salaam Tanzania; e Department of Pediatrics/Child Health, Muhimbili National Hospital (Mloganzila), Dar es Salaam Tanzania; f Department of Pediatrics/Child Health, Msambweni County Referral Hospital, Kwale Kenya; g H_3_ Clinical Research Unit, I-Katch Technology Ltd., Dar es Salaam Tanzania

## Abstract

**Background:**

Anaemia is a global public health indicator of both poor nutrition and poor health. Besides, it stands as a silent signal of mal-aligned health system across the entire human lifespan. Globally, the importance of anaemia is most pronounced among children. This study was conceived to assess severity and morphological characteristics of anaemia among children aged from 6 to 59 months old in Temeke, Dar es Salaam, Tanzania.

**Methods:**

We designed a cross sectional, clinics-based analytical study. Children aged 6 to 59 months with anaemia were the target population. Severity and morphological characteristics of anaemia were the main outcome variables. Data were collected using a pre-designed questionnaire. Data were summarised using median and inter-quartile range (continuous variables) or frequency and proportions (categorical variables). Chi-square tests were applied to assess association between categorical variables. Alpha level of 5% was used as a limit of type 1 error in findings. Written informed consent was sought from mother of each child prior to inclusion into the study.

**Results:**

We successfully recruited and analysed 250 children. Participants median age was 17.5 (IQR: 9 - 34) months (females, n=112, 44.8%). Point prevalence of anaemia (Hb<12 g/dL) was 66.8%. Among anemic children (n=167), about 19%, 63% and 18% had mild, moderate and severe anaemia respectively. A direct linear association between MCV and MCHC was observed among anemic children (n=167, Spearman's rank ´Y= 0.86, *P=.000*). There was a significant association between prevalence and severity of anaemia among studied children (LR χ^2^ (corrected) = 229.5, df=3). Majority (n=121, 72%) of the studied children had normocytic normochromic anaemia.

**Conclusion:**

Majority of under-fives in attendance at outpatient clinics in Temeke were anemic. Normochromic normocytic anaemia was the most prevalent variant of anaemia in this study population.

## BACKGROUND

Anaemia is a significant secondary clinical diagnosis in children under the age of five, especially in developing countries.^1,2^ Globally, the public health importance of anaemia is by far most consequential in children (especially under-5 years).^2^ It is associated with various health problems such as recurrent infections, delayed developmental milestones, poor academic performance, and increased morbidity and mortality.^1,3-5^ However, besides all the accumulated evidence, the relationship is not evidently justified to be that of cause-effects.^6^ Furthermore, anaemia in children has been associated with mortality.^7^ Moreover, unlike anthropometric indices, anaemia in children is hard to accurately measure, particularly in limited resources settings.^8^ WHO – SARA 2020 reported that 57% of health facilities only could test hemoglobin levels among children in Tanzania.^9^ Moreover, about 68% of the facilities were reportedly offering iron supplementation in children.^9^ There is substantial evidence, that Tanzania central government's initiatives, to solve potential challenges, responsible for under 5-year mortality, to include <5-years anaemia. It was thus of particular interest for investigators, to assess the pattern of anaemia, in terms of its morphology and severity among under-fives population, in typical urban Tanzanian settings.

WHO-SARA 2020 evidently reported lack of resources to diagnose Anaemia at an early stage.^6^ As a result, anaemia goes undetected and untreated at most primary health care facilities.^6^ The cascade of systemic failure to identify anaemia early on goes to management. WHO-SARA 2020 reported treatment with iron supplementation to every child with anaemia, irrespective of causes of anaemia.^6^ The immediate observation from the findings, suggest an obvious gap, in severity and morphological burden of anaemia, especially among under-fives children. Morphological patterns of anaemia in children aged 6 to 59 months are essential for classification, diagnosis, and management. They are also important for assessing the morphology of RBCs as the clinical features in infants and preschool children could aid in determining the cause of anaemia and providing treatment to patients.^7, 8^ However, to date, there is no evidence in retrievable published literature that accounts for severity and morphological patterns of anaemia among under-fives in Dar es Salaam, Tanzania.

Temeke demographic health survey data reported anaemia among top ten conditions that affect children less than five years.^9^ The revelations comes despite efforts done by the government in the prevention and treatment (e.g. deworming <5-years old children, iron supplementation), still anaemia has 9% mortality rate, with increased flow of children in outpatient and inpatient departments by 10% and 4% respectively^9^ That calls for a potential gap on possibilities of ‘non-iron deficiency anaemia’ among this target population. However, there is no evidence of solid data retrievable in peer-reviewed platforms on the topic. At global scale, anaemia is reported to exist in children aged 6-59 months when circulating red blood cells and/or hemoglobin concentration are insufficient to meet physiological oxygen-carrying needs.^8^ Specifically, children aged 6-59 months are reportedly to have mild, moderate or severe anaemia if their estimated blood hemoglobin concentration levels are 10-10.9g/dL, 7.0 – 9.9g/dL or <7g/dL respectively.^8^ At present, there is absence of retrievable data on causes of anaemia in children in Temeke. We thus, hypothesized that anaemia among <5-years children in Temeke to be unrelated to ‘iron deficiency’. The hypothesis of which called for a baseline data to substantiate nature and probable causes of anaemia in this otherwise important population group.

## MATERIALS AND METHODS

### Study Area

The study was conducted at Temeke regional referral hospital and Mbagala Rangi Tatu district hospital. The two hospitals serve as public healthcare facilities for Temeke municipality in Dar es Salaam metropolitan city. Temeke is the largest of all the three official Dar es Salaam municipalities. According to official data of 2020-2021, Temeke had the poorest scores in the indices of anaemia among <5-years children and pregnant women of all Dar es Salaam municipalities.^9^ Temeke regional referral hospital is the highest referral public health facility in Temeke municipality of Dar es Salaam city. Moreover, Mbagala rangi tatu district hospital serves as among public district referral health facilities in Temeke. By far, it is the only public referral health facility covering the original Temeke before the merge of Temeke and Kigamboni areas making Temeke region in the local health system. Temeke regional referral hospital is found at -6.86008 S and 39.26256 E. Mbagala rangi tatu district hospital is found at -6.91422 S and 39.2708 E.

### Study Design, Duration and Population

A cross sectional clinics-based analytical study was done in July-December 2022. We included and screened all children for both anaemia as well as determining their severity and morphological patterns during a single visit. Our target population was children aged 6-59 months old. Details about this study design has been reported before.^10^

### Sampling

All children aged 6-59 months attended at the two study settings, officially registered on outpatients basis, at either Mbagala Rangi Tatu district hospital or Temeke Regional Referral Hospital during the study period were eligible to participate into the study. The exercise was achieved via adoption of random number generators from MS-Excel, and confer them to clinic attendees during the study duration until a desirable sample size was reached. However, that planned activity was later on considered to include all children since the study investigators managed to recruit all children. Moreover, Cochran formula was used to determine the minimum sample size.^13^ In order to account for some extraneous activities, an additional 10% of the minimum sample size value was recruited in order to account for missing data as well as non-participants into the study for the same demanded study power of at least 80%. Thus, a minimum sample size of around 198 (with additional 20 children) was needed to answer the research question while maintaining a study power of 80%, an α-level of 5% as a limit of type 1 error in findings while keeping the effect size at around 10%. Initial estimates of anaemia among <5-year old at Temeke was estimated at 31.4% back in 2021-2022 data.^9^

### Recruitment Criteria

The study population was eligible to be included in the sample provided that they were aged between 6-59 months and attended as outpatients at either Temeke regional referral hospital or Mbagala rangi tatu hospital. Besides, they must had neither received blood transfusion in the past 120 days nor were they supposed to be evidently given any intravenous fluid infusion/hematenics at least 14 days prior to the recruitment date. Otherwise, a simple random sampling technique was used to obtain the minimum sample size needed to achieve the study power to answer the study question.

### Data Collection Tool

An interviewer administered questionnaire with five distinct parts was the main tool for data collection. The questionnaire was developed in English and then translated into Swahili language. Part A of the questionnaire consisted of baseline demographic data of the child and parents/guardians. Part B consisted of symptoms of anaemia including (but not limited to) fever, general body weakness, yellowish staining of the eyes, paleness of hand and feet, anorexia, cough, pica, and swelling of the body. Part C was comprised of physical signs of anaemia, including (but not limited to) pallor, jaundice, hepatomegaly, splenomegaly, edema, lymphadenopathy and the sign of congestive heart failure. Part D consisted of anthropometric measurements of weight, height, and length. Part E comprised of of lab parameters from a sterile collection of samples using 70% ethanol, a sterile needle, and a 5cc syringe, and placing them in a vacutainer test tube for a full blood count.

### Data Collection Process

Data collection was performed primarily by a Principal Investigator (PI) and two research assistants (one practitioner and a laboratory technologist). Both were trained on Good Clinical Practice (GCP) for a day workshop prior to undertaking the research functions. Children recruited were identified in outpatient pediatric clinics at the two health facilities and after written informed consent from parents/guardians. Immediately following written informed consent from parents/guardians data were collected by interviewing mothers/guardians of children aged 6 to 59 months, attending outpatient clinics at either Temeke Regional Referral Hospital or Mbagala rangi tatu district hospital using the pre-designed and pre-tested structured questionnaires. About 2-3mls of blood was drawn from the left antecubital fossa or femoral area of the left foot of each studied child. Thereafter, the Principal Investigator and research assistants used a pre-designed and pre-tested structured questionnaire for interviews and a complete physical examination was performed immediately afterwards. The axillary body temperature of each child was measured using a digital thermometer and fever was defined as a temperature ≥ 37.5 °C. Anthropometric measurements done included height, length, and weight. They were measured using a Stadiometer and weighing machine (Momerta (TM) Co.Ltd, Hungary) respectively. Anthropometric measurements were intepreted per WHO standards with under-nutrition indices that comprised weight-for-height / length (WH/L) standard deviation (SD) scores (z-scores) computed based on the World Health Organization questionnaires. The questionnaire specifically comprised of the three initials of the name, child age in terms of months, +/- reported history of chronic illness(es) and sign and symptoms (like fever, general body malaise, fatigue, yellowish discoloration of eyes and heart palpitation, paleness, jaundice, lymphadenopathy, hepatomegaly). Laboratory investigations to determine hemoglobin level and red blood cell indices were analysed using automated hematology analyzer, DYMIND co. limited model DH 76 (Abbott Laboratories, USA) PI and research assistants explained the procedure of sample collection to the mother/guardian. Following applied tourniquet proximal to the site of venipuncture, using an aseptic technique after disinfection with cotton immersed in 70% ethanol, 2-3 milliliters of venous blood was collected, either from ante-cubital fossa or proximal femoral vein by using a 2-5 cc syringe. The samples were subsequently collected in EDTA vacutainer, and stored in cold box container, transported from Mbagala Rangi tatu to Temeke Regional Referral Hospital by motorcycle for full blood picture analysis. PI and two research assistants (one is a laboratory technologist - a degree holder in medical laboratory sciences; and the second one a principle clinical officer) completed the task. PI and other investigators were responsible to train research assistants on how to collect samples by using standard operating procedures. A laboratory technologist at Temeke Regional Referral Hospital was tasked to analyze all blood samples.

### Data Analysis

Immediately following data collection, all data in questionnaire were double entered into a pre-designed SPSS software version 23 (SPSS (TM) IBM SPSS Statistics, USA) template, cleaned (checking for any incomplete data, errors in data entry) and later stored in the laptop computer of the Principal Investigator until analysis time.

Data analysis commenced initially with data exploration and summarisation of important variables of interest. Specifically, continuous data was summarised using median (with inter-quartile range) and categorical data was summarised using frequency (and proportion by %) Graphical tools were also employed, where by important correlations as well as +/- outliers were assessed for their significance in primary findings. Besides, for assessing association between different categorical variables, a likelihood ratio chi-square test statistics with corresponding degrees of freedom was used.

A complete blood count - a medical laboratory finding that shows the number of different blood parameters, including white blood cells, red blood cells counts, platelets, hemoglobin concentration, hematocrit and red blood cells morphology was also assessed. Anaemia was reported based on hemoglobin levels and red blood cell indices; and anaemia was defined based on WHO criteria, with hemoglobin levels less than 11g/dl being mild, less than 10g/dl being moderate, and less than 7g/dl being severe; and morphology of anaemia was reported based on red blood cell indices, with mean corpuscular volume of 70-98 fl being normocytic anaemia, less than 70 fl is microcytic anaemia and more than 98 fl is macrocytic anaemia. Mean cell hemoglobin concentration is classified as normochromic, hypochromic, or hyperchromic.^14^ We planned (during design) to include peripheral smear analyses but the service was not available at all studied facilities. For those who were found to have severe anaemia (Hb<7g/dl) and sepsis were admitted for further evaluation and management, including blood transfusion, and were followed-up via the routine standard operating procedures at health facilities. They were given priority as severe anaemia and sepsis are life threatening conditions if left untreated.

### Dependent Variables

Hemoglobin level and red blood cell indices of RBC counts, MCV, MCHC and MCH.

### Independent Variables

Age, sex, residence, child anthropometric measurements (weight for height/length), +/- fever, +/- loss of appetite, +/- paleness, +/- jaundice, +/- lymphadenopathy, +/-hepatomegaly, and +/- splenomegaly.

### Ethical Considerations

Ethical clearance to conduct this study was obtained from the Institutional Research Ethics Committee of Hubert Kairuki Memorial University. Permission to undertake the study at the study sites was sought from the administrations at Temeke Regional Referral Hospital (TRRH) and Mbagala rangi tatu (via the office of the municipal director at Temeke municipal council) district hospital.

## RESULTS

The study recruited and analysed 250 children aged 6 to 59 months who were attended Temeke regional referral hospital and Mbagala rangi tatu district hospital. Consequently, 167 (66.8 %) children were found to be anemic. The baseline demographic characteristics of study participants are as indicated in Table 1.

**Table 1.** Selected Baseline Demographic Characteristics of Children Aged 6 to 59 Months Attended at Temeke Municipal Facilities, Dar Es Salaam

Continuous Variable	Median	Inter-Quartile Range
Age (in completed months)	17.5	9 – 34

Categorical Variable	Frequency	Proportion (%)
Sex		
Male	138	55.2
Female	112	44.8
Total	250	100
Permanent Residence		
Temeke	193	77.2
Outside Temeke	48	19.2
Outside Dar-es salaam	9	3.6
Total	250	100

Likewise, investigators managed to collect some selected clinical characteristics of all analysed children. Clinical characteristics of studied children are as summarised in Table 2.

**Table 2.** Summary of Clinical Characteristics of Children Aged 6 to 59 Months Attended Paediatric Outpatient Clinics at Temeke Municipality, Dar Es Salaam

Continuous Variable	Median	Inter-Quartile Range
Haemoglobin level (in g/dL)	10.1	8.5 – 11.6
Mean Cell Volume (fL)	86	77 – 93
Mean Cell Haemoglobin (pg/cell)	26	22 – 29
Mean Cell Haemoglobin Concentration (g/dL)	29	27.8 – 31.1

Categorical Variables	Absolute Counts	Proportion (%)
Anaemia status		
Anemic (Hb<12g/dL)	167	66.8
Normal Hb level (Hb: 12 – 17g/dL)	83	33.2
Nutritional status		
Normal	131	52.4
Moderate Acute Malnutrition	22	8.8
Severe Acute Malnutrition	7	2.8
Overweight	43	17.2
Obesity	26	10.4
Severe Obesity	21	8.4

**Figure 1.**
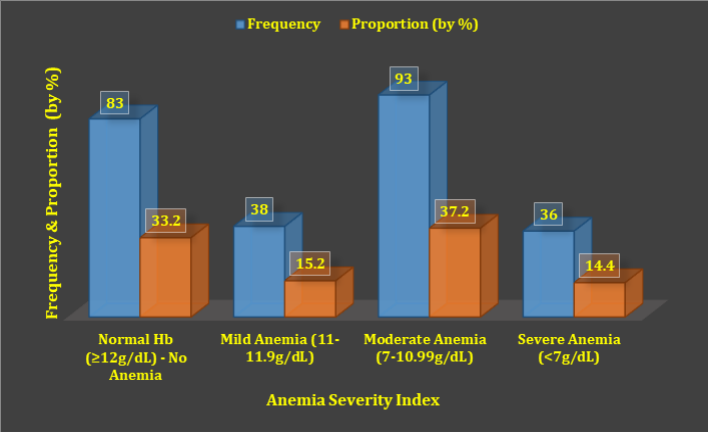
Frequency Distribution By Severity Of Anaemia Among Children Aged 6 to 59 Months Attended at Temeke Referral Facilities **Note:** LR-Chi-square value (corrected) = 229.5, df = 3 (*p=.000*)

It was also of interest to assess severity of anaemia among study participants. Figure 1 summarises hemoglobin levels by severity of anaemia among studied children.

**Figure 2.**
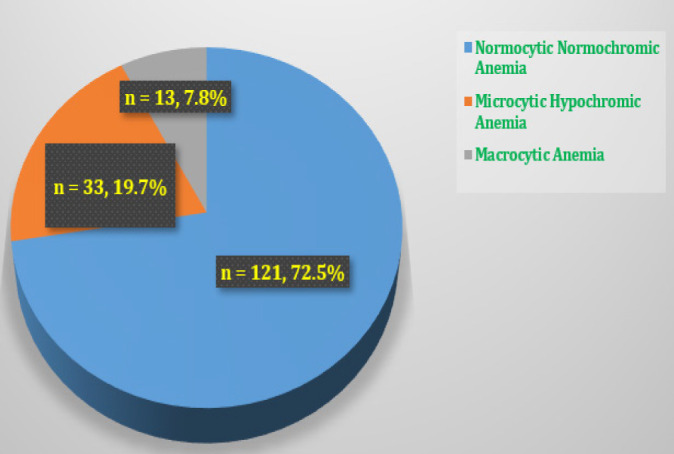
Morphological Classification Of Anaemia Among Children Aged 2-59 Months Attended At Pediatric Outpatient Clinics In Temeke Referral Hospitals – Dar es Salaam

Investigators also had interest to characterize morphological characteristics of anaemia among studied children. Table 3 summarises the morphological characteristics of anaemia among study participants Hematological indices of each studied participant was analysed. Six different morphological types of anaemia were identified as described in Table 3, but in Figure 2 the most common morphological classification is described.

**Table 3.** Morphological Classification of Anaemia Among Children Aged 6 To 59 Months Attended Paediatric Opd At Temeke Referral Facilities (July – Dec 2022)

MCV (fl)	MCHC Hypochromic (< 32 g/dl)	(g/dl) Normochromic (32-36g/dl)	Total
Microcytic (< 70fl)	33	0	33
Normocytic (70-98 fl)	0	121	121
Macrocytic (>98 fl)	0	13	13
TOTAL	33	134	167*

Note: Spearman's rank correlation coefficient=0.86, *P=.000*.

* We only included children (n=167) with anaemia (Hb values <12g/dL).

We managed to get clues to the probable causes of anaemia in 31 (18.5%) of the studied children. Of which 18 tested positive for malaria, 7 had positive sickling test (and confirmed with Hb-electrophoresis) and six children had worm infestations in their stools. For the rest, of the children, no potential possible/probable cause(s) of anaemia was evident.

## DISCUSSION

We found two thirds (66.8%) of all studied children to had been anemic. Given such an estimate, anaemia in this specific population group seems to be a fairly public health problem in Temeke municipality. This calls for special attention for primarily two reasons. First (and perhaps the foremost) Temeke municipality is the biggest of all in Dar es Salaam city. Dar es Salaam city being the business capital has been enjoying the *lion's share* in national health expenditures. The figures therefore suggest health care expenditures may not be congruent to expectations, as far as SDG 2030 indicators are concerned. Second, Temeke is largely an industrial zone of not only Dar es Salaam city but also entire Tanzania. Most food processing industries are found in Temeke. Besides, some of those food industries and factories include elements of micronutrient fortification (including iron and folic acid). Thus, Temeke figures of under-fives anaemia may reflect a latent signal of a potentially alternate challenge than the ones already evident in literature as far as anaemia and iron deficiency are concerned. At a gross scale, these figures call for an immediate intervention since they completely antagonize value-for-money *design thinking* approaches in healthcare expenditures as far as evidence-based clinical and public health approaches are concerned.

The current study findings on gross anaemia among under-fives population at Temeke are comparable to other findings in Dar es Salaam and Tanzania published before.^11, 15-18^ For instance, Tanzania Demographic and Health, Malaria Indicator Survey of 2016 revealed an estimated prevalence of any anaemia to the magnitude of around 58% among <5 years old children.^11^ Likewise, Simbauranga and others reported a prevalence of 77.2% for anaemia among 448 under-five year old children in a cross-sectional study from Mwanza region, Tanzania between 2012 and 2013.^15^ However, that study by Simbauranga and her colleagues was designed using inpatients cohort from pediatric department at Bugando Medical Center; which is a zonal referral hospital. Children admitted to a zonal referral facility are likely to be sicker, complicated by more than 1 comorbidities, and in advanced stages of their illnesses compared to our study population comprising of an otherwise stable outpatients' population base only.

Moreover, another study by Fabian Mghanga found the prevalence of anaemia of about 83% among under-fives admitted to a referral facility in the Southern parts of Tanzania.^15^ However, their study population included inpatient children only; and the study was a retrospective patients' file-based by design.^15^ It is equally likely the difference in prevalence to ours, to had been partly attributed by the nature of the sample (inpatients only) and/or study design (retrospective file based). Data from retrospective design may be biased since often times, what gets analysed is what was retrieved. What so ever, estimates are likely to differ dependent on the file keeping strategies rather than true burden/status of the condition. On a gross scale however, all the cited studies (including our current findings) provide a snapshot for the potential public health calamity among under-five year old children throughout Tanzania. Furthermore, should we consider effects of chronic anaemia in childhood to be reflected throughout the entire human lifespan,^19-21^ the current study findings are probably a wake-up call for immediate state interventions in our study settings. Dar es Salaam is set to be the 10^th^ most populated city on earth, and the 3^rd^ in Africa come 2050.^22^ Besides, there are significant evidence that Tanzania is ageing at a relatively faster rate than most other African countries.^22^ What is likely a direct connection to the current study being evidence that childhood adversity (especially infections and anaemia) predispose to later age inflammatory conditions,^21-25^ and through different mechanisms (known and unknown) that are likely to be chronic and non-communicable over time.^26-32^ Thus, intervening childhood anaemia serves double benefits to both current and future maladies. Therefore, childhood adversity of this nature may warrant special attention, due to their potentiality towards havoc at a population level for the foreseeable future. There is little data to link what happened to majority of adult and senior Tanzanians but yet, evidence is clear of their ill-health.^24, 25^ Besides, reliability and validity of information published, especially to connect childhood adversity and later life debilitating conditions have been questioned in literature.^26^ Against all odds, we considered our study to be the probable most referent near-population based to the Dar es Salaam children at present. We hope future studies on the agenda to be derived from population based surveys in order to yield more representative estimates of this otherwise important clinical and public health parameter.

A significant proportion (63%) of studied children had moderate anaemia. These findings are comparable to others published before, both from within^15,16^ and outside Tanzania.^26-28^ The systematic review on global, regional and national estimates of anaemia among <5 years children revealed the global prevalence of moderate anaemia among <5 years to had been 42%.^27^ A previous study done in Dar es Salaam about 40 years ago revealed the prevalence of anaemia among under-six years to had been 50%.^18^ It is still ill-understood whether the current findings signified a probable reduction in the trend for anaemia prevalence across the population groups or a mere continuation of the same. In Kimati and colleagues paper, they included children under 6 years of age that were admitted at the highest referral facility in the country.^18^ It is highly likely that children admitted at the highest referral point to be composed of sicker children, and hence their anaemia state to be significantly higher compared to those found in lower referral centers and community settings like in this study. Thus, future studies on this topic will benefit scholars, policy makers and clinicians if efforts will be made to incorporate children across different referral points.

Furthermore, we have reported majority (72%) of ‘children to had been normocytic normochromic anemic; followed by microcytic hypochromic anaemia (13.6%). Macrocytic anaemia (5.2%) had the least presentation. To the best of our knowledge, when these findings are analyzed on the lens of the fact that most of the studied children were generally anemic, with at least simple majority post their infancy period, the picture of probable chronic states of anaemia cannot be ruled out. In fact, it has been evidently documented before in Tanzania.^30^ However, we wish to consider that as a speculation, since our study was limited by design from causality modeling for anaemia. We are hesitant to assume the cause to be ‘non-iron deficient anaemia’ due to a number of likely confounding factors in operation (e.g. acute iron deficient state?, erroneous intervention with iron tablets??) Furthermore, as a shortcoming to the current work, we could estimate the probable causes of anaemia in about a fifth of the studied children only. Besides, in our current study, the design was cross-sectional and clinics-based (outpatients) and hence we were unable to account for a number of other important social and environmental traits. Moreover, we could not account the possible temporal association by virtue of our study design. Notably, our analyses on morphological patterns of RBCs were also weak since we could not get peripheral smear tests. Thus, from epidemiological standpoint, and on the basis that anaemia is a secondary clinical diagnosis to a number of other myriad of clinical conditions, future studies on this topic need to account potential etiological factors with possible interventional designs. For that to be possible, they need to be designed in a prospective longitudinal manner. However, our study included all children managed at the facilities during the study period. That served as an added advantage since our estimates are unlikely to be influenced by the draw of chance or biases associated with both random and non-random samples in observational studies.

## CONCLUSION

Anaemia was prevalent among under-five years old attending outpatient referral clinics in Temeke municipality.

Moderate anaemia dominated the clinical picture in this study population. Normochromic normocytic anaemia was the most dorminant morphological pattern found in the studied children.

## References

[1] Walker S, Wachs T, Gardner J, Lozoff B, Wasserman G, Pollitt E, et al. Child development: risk factors for adverse outcomes in developing countries. Lancet 2007; 369 (9556): 145–157. 17223478 10.1016/S0140-6736(07)60076-2

[2] Editorial. Anaemia: a child health indicator we cannot neglect. Lancet Child & Adolescent Health 2023; 7(7): 441. 37349015 10.1016/S2352-4642(23)00147-5

[3] Pollitt E, Leibel R. and Greenfield D. Iron deficiency and cognitive test performance in preschool children. Nutrition & Behavior 1983; 1(2): 137–146.

[4] Soewondo S. The effect of iron deficiency and mental stimulation on Indonesian children's cognitive performance and development. Kobe J Med Sci. 1995; 41 (1-2): 1–17. 7490909

[5] Lozoff B, Brittenham G. and Wolf A. Iron deficiency anaemia and iron therapy: effects on infants development test performance. Pediatrics 1987; 79: 981–995. 2438638

[6] Grantham-Mc Gregor S. and Ani C. A review of studies on the effect of iron deficiency on cognitive development in children. J Nutr. 2001; 131(2): 649S - 668S. 11160596 10.1093/jn/131.2.649S

[7] Lozoff B, Jimenez E. and Wolf A. Long-term developmental outcomes of infants with iron deficiency. N Engl. J. Med. 1991; 325: 687–694. 1870641 10.1056/NEJM199109053251004

[8] Pasricha S-R, Colman K, Centeno-Tablante E, Garcia-Casal M-N. and Pena-Rosas J-P. Revisiting WHO haemoglobin thresholds to define anaemia in clinical medicine and public health. Lancet Glob. Health 2022; 10: e627–e639.. 29406148 10.1016/S2352-3026(18)30004-8

[9] Temeke council. Health Profile - Temeke Municipal Council. 2017; Available from: http://www.temekemc.go.tz/storage/app/uploads/public/59d/ba8/62e/59dba862ed84d383294414.pdf

[10] Omary S, Kalabamu F, Fataki M, Salum S, Mohamed U, Kimaro J. and Leshabari K. Severity and morphological classification of anaemia among children aged 2-59 months in Dar es Slaam, Tanzania: a cross-sectional study protocol. MedRxiv 2022. Available at https://www.medrxiv.org/content/10.1101/2022.11.10.22282169v1 [Accessed on 1 Feb 2024] 10.24248/eahrj.v8i2.780 PMC11407129 39296774

[11] Tanzania Ministry of Health. Tanzania Demorgraphic and Health Survey Malaria Indicator Survey (TDHS-MIS) 2015-2016. Dar es Salaam, Tanzania, Rockville, Maryland, USA MoHCDGEC, MoH, NBS, OCGS, ICF. 2016;1(1):1–630.

[12] National Bureau of Statistics. Tanzania in figures 2012. Available from www.nbs.go.tz›Tanzania_in_figures2012. [Accessed on 10 Jan 2023]

[13] Charan J, Kaur R, Bhardwaj P, Singh K, Ambwani SR, Misra S. Sample Size Calculation in Medical Research: A Primer. Ann Natl Acad Med Sci [Internet]. 2021 Apr 13;57(02):074–80. Available from: http://www.thieme-connect.de/DOI/DOI?10.1055/s-0040-1722104

[14] Stevens G, Finucane M, De-Regil L, Paciorek C, Flaxman S, Branca F, et al. Global, regional, and national trends in haemoglobin concentration and prevalence of total and severe anaemia in children and pregnant and non-pregnant women for 1995-2011: a systematic analysis of population-representative data. Lancet Glob Health 2013; 1(1): e16–e25. 25103581 10.1016/S2214-109X(13)70001-9 PMC4547326

[15] Mghanga FP, Genge CM, Yeyeye L, Twalib Z, Kibopile W, Rutalemba FJ, et al. Magnitude, Severity, and Morphological Types of Anaemia in Hospitalized Children Under the Age of Five in Southern Tanzania. Cureus [Internet]. 2017 Jul 21;9(7). Available from: http://www.cureus.com/articles/8029-magnitude-severity-and-morphological-types-of-anaemia-inhospitalized-children-under-the-age-of-five-in-southern-tanzania 10.7759/cureus.1499 PMC5608490 28948119

[16] Simbauranga R, Kamugisha E, Hokororo A, Kidenya B. and Makani J. Prevalence and factors associated with severe anaemia amongst under-five children hospitalised at Bugando Medical Centre, Mwanza, Tanzania. BMC Hematology 2015; 15:13. 26464799 10.1186/s12878-015-0033-5 PMC4603816

[17] Kejo D, Petrucka P, Martin H, Kimanya M. and Mosha T. Prevalence and predictors of anaemia among children under 5 years of age in Arusha district, Tanzania. Pediatric Health, Medicine & Therapuetics 2018; 9: 9–15. 10.2147/PHMT.S148515 PMC5804135 29443328

[18] Kimati VP, Lema RA, Magessa PM, Kumar KA. Childhood anaemias in Dar-es-Salaam. J Trop Pediatr. 1986;32(5):263–7. 3795339 10.1093/tropej/32.5.263

[19] Palti H, Pevsner B. and Adler B. Does anaemia in infancy affect achievement on developmental and intelligence tests? Hum Biol. 1983; 55(1): 183–194. 6840745

[20] Lozoff B, Jimenez E, Hagen J, Mollen E. and Wolf A. Poorer behavioural and developmental outcomes more than 10 years after treatment for iron deficiency in infancy. Pediatrics 2000; 105(4): E51. 10742372 10.1542/peds.105.4.e51

[21] Gilbert L, Breiding M, Merick M, Thompson W, Ford D, Dhingra S, et al. Childhood adversity and adult chronic disease: an update from ten states and the District of Columbia, 2010. Am J Prev. Med. 2015; 48(3): 345–349. 25300735 10.1016/j.amepre.2014.09.006

[22] Leshabari K. Demographic transition in sub-Saharan Africa: From grassroots to ivory towers. In Klimczuk A (ed). Demographic analysis: selected concepts, tools and applications. IntechOpen 2021; doi: 10.5772/intechopen.98407. Available from 10.5772/intechopen.98407 [Accessed on 23 Dec 2022]

[23] Leshabari K, Biswas A, Gebuis E, Leshabari S. and Ohnishi M. Challenges in morbidity and mortality statistics of the elderly population in Tanzania: a call to action. Quality in Ageing and Older Adults 2017; 18(3): 171–174. doi: 10.1108/QAOA-09-2016-0035. Available from https://www.emerald.com/insight/content/doi/10.1108/QAOA-09-2016-0035/full/html [Accessed on 1 Dec 2022]

[24] Leshabari K, Ndonde W, Mbega S. and Mbululo L. Predictors of cardiometabolic risks among the typical African elderly population: analysis from Dar es Salaam, Tanzania. Diabetes Technology & Therapeutics 2020; 22: A214.

[25] Chale G, Salim R. and Leshabari K. Clinical indications for total abdominal hysterectomy among women seen in Dar es Salaam regional referral hospitals, Tanzania: a prospective, observational hospital-based study. Pan African Medical Journal 2021; 38(10: 10.11604/pamj.2021.38.10.17695. Available from https://www.panafrican-med-journal.com/content/article/38/10/full/PMC7825369 33520079

[26] Leshabari K. Reliability and validity of clinicopathological features associated with frailty syndrome in elderly population. In Palermo S. (ed) Frailty in the elderly: understanding and managing the complexity. IntechOpen 2021; doi: 10.5772/intechopen.93499. Available from https://www.intechopen.com/chapters/73143

[27] World Health Organisation. Worldwide prevalence of anaemia 1993-2005. Available from https://www.who.int/publications/i/item/9789241596657. [Accessed on 14 October 2023]

[28] Alamneh Y, Akalu T, Shiferaw A. and Atnaf A. Magnitude of anaemia and associated factors among children aged 6-59 months at Debre Markos referral hospital, Northwest Ethiopia: a hospital cross-sectional study. Italian Journal of Pediatrics 2021; 47: 172. 34389033 10.1186/s13052-021-01123-3 PMC8362241

[29] Ocan A, Oyet C, Webbo F, Mwambi B. and Taremwa I. Prevalence, morphological characterisation, and associated factors of anaemia among children below 5 years of age attending St. Mary's hospital Lacor, Gulu District, Northern Uganda. Journal of Blood Medicine 2018; 9: 195 - 201. 30464670 10.2147/JBM.S184126 PMC6217135

[30] Leshabari K. and Ramji R. The pattern of infections among under-fives: a call for actions. Dar es Salaam Medical Students' Journal 2008; 15(1): 24–28. Available from https://www.ajol.info/index.php/dmsj/article/view/49597 [Accessed on 1 January 2023]

[31] Dover G. The Barker hypothesis: how paediatricians will diagnose and prevent common adult-onset diseases. Transaction of the American Clinical and Climatological Association 2009; 120: 199–207. PMC2744561 19768178

[32] Margolis R. Childhood morbidity and health in early adulthood: Life course linkages in a high morbidity context. Adv. Life Course Res. 2010; 15(4): 132–146. 21516232 10.1016/j.alcr.2010.10.001 PMC3079227

